# Erectile Function Decline in Men with Chronic Kidney Disease: A Three-Year Case–Control Study Comparing Haemodialysis, Non-Dialysis CKD and Community Controls

**DOI:** 10.3390/jcm15041402

**Published:** 2026-02-11

**Authors:** Merkourios Kolvatzis, Apostolos Apostolidis, Fotios Dimitriadis, Evangelos N. Symeonidis, Michael Samarinas, Konstantinos Hatzimouratidis, Kyriakos Moysidis

**Affiliations:** 1 2nd Department of Urology, Medical School, Aristotle University of Thessaloniki, 54124 Thessaloniki, Greece; merkolv90@gmail.com (M.K.); zefxis@yahoo.co.uk (A.A.); mikesamih@hotmail.com (M.S.); promahon@otenet.gr (K.H.); 2 1st Department of Urology, Medical School, Aristotle University of Thessaloniki, 54124 Thessaloniki, Greece; helabio@yahoo.gr; 3 Department of Urology II, European Interbalkan Medical Center, 55535 Thessaloniki, Greece; evansimeonidis@gmail.com

**Keywords:** erectile dysfunction, chronic kidney disease, haemodialysis, sexual function, IIEF-15, health-related quality of life

## Abstract

**Background/Objectives:** Sexual dysfunction is highly prevalent in men with chronic kidney disease (CKD), but longitudinal data across the CKD spectrum, particularly those directly comparing non-dialysis CKD with haemodialysis, are limited. We aimed to characterise longitudinal patterns in erectile and broader sexual function over three years, focusing on persistent between-group stratification and change over time in men with CKD versus community controls, and to identify clinical predictors of poorer outcomes. **Methods**: We conducted a three-year prospective cohort study in three groups of adult men: a group on haemodialysis, a group with non-dialysis CKD stages 3A/3B, and age-matched community controls without known kidney disease. The primary endpoint was the erectile function (EF) domain score of the International Index of Erectile Function (IIEF-15), assessed annually; the IIEF-15 total score and remaining domains were the secondary outcomes. Participants’ health-related quality of life (EQ-5D-5L), age, and diabetes status were recorded. Linear mixed effects models with participant-level random intercepts estimated the effects of group, year, and group × year, adjusted for age, EQ-5D-5L, and diabetes. **Results**: We enrolled 267 men (haemodialysis n = 96; CKD n = 88; and controls n = 83). At every time point, EF and other IIEF-15 domain scores showed a graded pattern with controls being the highest, CKD being intermediate, and haemodialysis the lowest. group × year interactions were not significant, indicating parallel trajectories without differential decline between groups over three years. Having a lower EQ-5D-5L, an older age, and diabetes—particularly type 2—were independent predictors of poorer IIEF-15 scores across domains. **Conclusions**: Male sexual function in CKD is persistently and gradually impaired along the renal disease spectrum, with patients on haemodialysis faring the worst and with no evidence of divergent longitudinal change. Routine EF screening, systematic attention to patients’ quality of life, and aggressive management of diabetes should be embedded in CKD care pathways, and renal-appropriate erectile dysfunction interventions should be considered earlier and more systematically.

## 1. Introduction

Erectile dysfunction (ED) is common across the adult male lifespan and increases with age, but its clinical relevance extends beyond sexual health: ED tracks closely with cardiometabolic morbidity and overall male health status [1,2,3,4]. Population studies in community samples have quantified the burden and demonstrated clear age gradients, while longitudinal work has confirmed incident ED in mid-life and late adulthood [5,6]. Meaningful proportions of new presenters are younger men, and modifiable factors such as adiposity, smoking exposure, poor glycaemic control and depressive symptoms are consistently implicated [7,8,9].

Chronic kidney disease (CKD) is a major non-communicable condition associated with excess cardiovascular and all-cause mortality and with substantial impairment in one’s health-related quality of life [10,11,12]. ED is highly prevalent across the CKD spectrum and is particularly frequent in men receiving haemodialysis; sexual dysfunction often predates dialysis initiation and persists during renal replacement therapy [13,14]. Pathophysiology in CKD is multifactorial, involving reduced nitric oxide bioavailability and endothelial dysfunction, diabetic autonomic neuropathy, hypogonadism with altered LH/FSH feedback, anaemia with impaired oxygen delivery to corporal tissue, medication effects and psychosocial factors [15,16,17,18]. These mechanisms co-occur and likely explain both the high burden and the heterogeneous treatment response. A recent state-of-the-art review across non-dialysis CKD, haemodialysis and kidney transplantation highlighted the persistent gaps in longitudinal, patient-reported outcome data [4].

The assessment of ED relies on validated patient-reported measures. The International Index of Erectile Function (IIEF-15) and its brief version (IIEF-5) quantify erectile function, orgasmic function, desire, intercourse satisfaction and overall satisfaction, and are standard endpoints in trials and observational research [19,20,21]. In nephrology populations, IIEF-based phenotyping typically shows graded decrements from community controls to non-dialysis CKD and the largest deficits in haemodialysis cohorts, with additional lowering among men with diabetes, mirroring the broader cardiometabolic phenotype of advanced kidney disease [12,22].

Most CKD studies to date are cross-sectional, providing important snapshots but limited insight into trajectories and timing of change [23,24]. Robust longitudinal data across CKD stages are scarce, constraining counselling, the timing of interventions and long-term planning. The distinctive contribution of the present work is its prospective longitudinal design with repeated IIEF-15 assessments across three annual time points in the controls, and in patients with non-dialysis CKD and haemodialysis. By modelling group and time, and their interaction, while adjusting for age, diabetes and health-related quality of life, we estimate covariate-adjusted longitudinal patterns rather than single-time contrasts. We hypothesised that baseline sexual function would be lower in CKD than in the controls and lowest in haemodialysis, and that men on haemodialysis would show a steeper decline over time, reflecting uremia-related vascular, neuroendocrine and treatment effects [14,25,26]. Our longitudinal framework differentiates this work by modelling within-patient changes over time and relating changes in sexual function to potentially modifiable clinical targets—dialysis adequacy, anaemia correction, medication review and ED-directed therapy—in line with contemporary nephrology and sexual–medicine recommendations [14,15,22,27,28].

## 2. Patients and Methods

### 2.1. Study Design and Population

This was a prospective, 3-year longitudinal observational case–control study (2023–2025) in three male cohorts: (1) those with pre-dialysis stage 3 CKD (KDIGO G3a/G3b; 30 ≤ eGFR < 60 mL/min/1.73 m^2^ by CKD-EPI), (2) those with end-stage renal disease (ESRD) on maintenance haemodialysis; and (3) controls without known kidney disease. Two case cohorts and one control cohort were enrolled in Thessaloniki, Greece. The ESRD cohort comprised men receiving thrice-weekly in-centre haemodialysis at the Renal Units of Papageorgiou General Hospital and Papanikolaou General Hospital. The CKD cohort comprised men with pre-dialysis CKD 3A/3B followed at the Nephrology Outpatient Clinic of Papageorgiou General Hospital. The controls were men attending the “General Urology” outpatient clinic of the 2nd Urology Department of the Aristotle University of Thessaloniki and additional community/outpatient sources (companions and flyer respondents), frequency-matched for age and without self-reported erectile dysfunction.

The eligible participants were adult males (≥18 years) who had been sexually active within the prior six months. The exclusion criteria were prior radical pelvic surgery, neurogenic erectile dysfunction and baseline use of ED-specific medications. Enrolment spanned a 12-month period, with annual evaluations for three years (Years 1–3). The analytic sample included 267 men (haemodialysis n = 96; CKD n = 88; and controls n = 83). The study was conducted in accordance with the Declaration of Helsinki and applicable national regulations on biomedical research and data protection. The protocol was approved by the Scientific Council of Papanikolaou General Hospital (Prot. No. 30/07-01-2019), the Scientific Council of Papageorgiou General Hospital, Thessaloniki (Prot. No. 9245/28-03-2022) and the Aristotle University Ethics Committee (Prot. No. 194/10/7/2023). All participants provided written informed consent before enrolment.

### 2.2. Data Collection and Variables

At baseline, we recorded age, body mass index (BMI), comorbidities (diabetes, hypertension, and cardiovascular disease), smoking status and current medications. In the CKD cohorts we documented CKD aetiology and stage/eGFR; in the haemodialysis group we additionally collected dialysis duration. A brief sexual history (prior ED or treatment) was also obtained. The same core variables and questionnaires were collected at each annual follow-up. Within the haemodialysis group, patients were stratified by dialysis duration into ≤12 months, 1–3 years, 4–7 years and ≥7 years. Within the CKD group, renal function was staged according to KDIGO criteria based on eGFR, with intermediate stages G3a (45–59 mL/min/1.73 m^2^) and G3b (30–44 mL/min/1.73 m^2^) represented.

### 2.3. Assessment Tools

The IIEF-15 [19,20,21] was the primary instrument for sexual function, covering erectile function (ef), orgasmic function, sexual desire, intercourse satisfaction and overall satisfaction. Higher scores indicate better function (total IIEF range 5–75; EF domain 1–30). For descriptive purposes, EF severity was categorised as follows: no ED: 26–30; mild: 17–25; mild-to-moderate: 11–16; moderate: 7–10; and severe: ≤6. Health-related quality of life was assessed at each visit using EQ-5D-5L.

### 2.4. Statistics

#### Outcomes and Study Sample Calculation

Primary and secondary outcomes were pre-specified. The primary outcome was the between-group difference in the longitudinal pattern of erectile function as measured by the IIEF-15 Erectile Function (EF) domain over follow-up. The secondary outcomes included (i) the IIEF-15 total score and the remaining subdomains (orgasmic function, sexual desire, intercourse satisfaction, and overall satisfaction), (ii) associations with age, health-related quality of life (EQ-5D-5L), and diabetes status, and (iii) clinically relevant baseline correlates recorded in the cohort.

Sample size planning was anchored to the IIEF-based primary endpoint and a conservative two-group contrast informed by the only closely comparable published study available at protocol development [29]. The final enrolment exceeded the minimum target and supported three-group longitudinal modelling. Specifically, we enrolled 267 participants (n = 96 haemodialysis, n = 88 non-dialysis CKD with eGFR < 60 mL/min/1.73 m^2^, and n = 83 controls), providing adequate power for between-group contrasts and improved precision for estimating time effects and group-by-time interactions within the mixed effects framework. The follow-up was complete (no attrition), and the IIEF-15 domain and total scores were available for all participants at each annual assessment; Supplementary Table S1 reports observed (unadjusted) summary statistics by group and time point.

### 2.5. Statistical Analysis

The continuous variables were inspected for distribution; non-normal data are summarised as median [Q1–Q3] and compared using Kruskal–Wallis tests (or ANOVA where appropriate). The categorical variables were presented as n (%) and compared using χ^2^ or Fisher’s exact tests. Two-sided α was set at 0.05; post hoc pairwise tests used Holm/Bonferroni adjustment. Longitudinal associations for the IIEF-15 total and each domain scores were estimated using linear mixed effects models with a participant-level random intercept to account for within-person correlation. The fixed terms were year (categorical: 1, 2, and 3), group (haemodialysis, CKD, and control), the group × year interaction, age, EQ-5D-5L, and diabetes status (type 1, and type 2). Fixed effect inference used Satterthwaite-type degrees of freedom. We report β, standard error, two-sided *p*-values and 95% confidence intervals. Global tests of the group × year interaction used Wald or F statistics. Estimated marginal means (EMMs and LS-means) were computed for each year × group cell, holding age and EQ-5D-5L at their sample means and diabetes at its modal level; pairwise contrasts were Holm/Bonferroni adjusted. Any missing outcome data were handled implicitly by the mixed model likelihood under a missing-at-random assumption; no outcome imputation was performed. As sensitivity analysis, we examined cluster-robust standard errors at the participant level. Analyses were performed in Python (version 3.11.8) using panDas (version 1.5.3), NumPy (version 1.24.0), SciPy (version 1.14.1), StatsModels (version 0.13.5), patsy (version 1.0.1) and Matplotlib (version 3.6.3).

## 3. Results

### 3.1. Baseline Characteristics

A total of 267 men were enrolled and allocated to three pre-specified groups: the haemodialysis group (n = 96), the non-dialysis chronic kidney disease (CKD) group with an estimated glomerular filtration rate (eGFR) < 60 mL/min/1.73 m^2^ (n = 88), and a control group without CKD (n = 83). Across the groups, participants were broadly similar in age distribution, with median (IQR) ages of 63.0 (53.0–70.0) years in haemodialysis, 67.5 (57.8–72.0) years in CKD, and 65.0 (55.0–70.5) years in controls; the omnibus comparison was not statistically significant (Kruskal–Wallis *p* = 0.1104).

In contrast, body mass index (BMI) differed materially by group (Kruskal–Wallis *p* < 0.0001). The participants on haemodialysis had the lowest median BMI at 24.85 kg/m^2^ (21.67–27.84), whereas participants with CKD had the highest median BMI at 27.09 kg/m^2^ (25.50–29.62), with controls being intermediate, at 26.60 kg/m^2^ (24.20–28.85). Post hoc pairwise tests (Bonferroni adjusted) indicated the significantly lower BMIs of patients on haemodialysis compared with CKD (*p* < 0.0001) and compared with controls (*p* = 0.0211).

Within the CKD stratum, renal impairment was confined to moderate CKD: 67.0% (59/88) were classified as KDIGO stage G3a and 33.0% (29/88) as stage G3b; no participants with G4–G5 CKD were recruited, defining the generalisability of the CKD findings primarily to stage 3 disease. In descriptive terms, eGFR in the CKD cohort had a mean (SD) of 47.73 (6.39) mL/min/1.73 m^2^ and a median (IQR) of 48.50 (43.00–52.00), spanning 33.00–58.00.

In the haemodialysis cohort, dialysis vintage was heterogeneous, with 15.6% (15/96) receiving dialysis for ≤12 months, 37.5% (36/96) for 1–3 years, 16.7% (16/96) for 4–7 years, and 30.2% (29/96) for ≥7 years.

Several sociodemographic variables differed between groups. Educational attainment varied significantly (χ^2^ *p* = 0.0106): tertiary education was common in the haemodialysis group (61.5%) and the controls (62.7%), whereas the CKD group most frequently reported secondary education (55.7%), yielding significant pairwise differences for the haemodialysis versus the CKD group (Bonferroni *p* = 0.0130) and CKD versus controls (Bonferroni *p* = 0.0360). Nationality also differed (χ^2^ *p* = 0.0337), although the sample was overwhelmingly Greek across the strata (Greek nationality was 92.7% in haemodialysis, 98.9% in CKD, and 100.0% in controls). Marital status differed (χ^2^ *p* = 0.0051): marriage was the most common status in all groups but was more frequent in the CKD (81.8%) and haemodialysis groups (71.9%) than in the controls (55.4%), with a significant post hoc contrast for CKD versus the controls (Bonferroni *p* = 0.0085). Parenthood did not differ significantly (χ^2^ *p* = 0.3338), with 79.2% of haemodialysis, 79.5% of CKD, and 71.1% of controls reporting children.

Employment status demonstrated the strongest sociodemographic separation (χ^2^ *p* < 0.0001). The participants on haemodialysis were predominantly retired (78.1%) and the participants with CKD were also commonly retired (64.8%), whereas the controls more frequently reported employment (48.2%). All three pairwise comparisons were significant after Bonferroni correction (haemodialysis–CKD, *p* < 0.0001; haemodialysis–controls, *p* < 0.0001; and CKD–controls, *p* = 0.0455).

Lifestyle and residential factors also varied. Smoking status differed (χ^2^ *p* = 0.0320): participants on haemodialysis more often reported current smoking (45.8%), whereas the CKD group most frequently reported former smoking (44.3%); the significant post hoc contrast was between haemodialysis versus CKD (Bonferroni *p* = 0.0253). The place of residence (urban versus rural) showed pronounced between-group differences (χ^2^ *p* < 0.0001). Urban residence was reported by 56.2% of the haemodialysis group compared with 85.2% of the CKD group and 79.5% of the controls, with significant post hoc differences for haemodialysis versus CKD (Bonferroni *p* < 0.0001), and haemodialysis versus controls (Bonferroni *p* = 0.0040).

Diabetes mellitus prevalence did not differ significantly at baseline across the three groups (χ^2^ *p* = 0.0793) when summarised using the categories presented in Table 1, supporting its interpretation as an important covariate rather than a primary differentiator between strata in this sample.

Relationship context and recent sexual activity also differed by group. Most participants reported monogamy, but the distribution varied (χ^2^ *p* = 0.0006): monogamy was almost universal in the CKD group (98.9%) compared with 88.5% in the haemodialysis group and 84.3% in the controls; post hoc differences were evident for the haemodialysis versus the CKD group (Bonferroni *p* = 0.0499) and CKD versus controls (Bonferroni *p* = 0.0014). The proportion reporting an ongoing sexual partner was high in all groups (85.4% in haemodialysis, 95.5% in CKD, and 85.5% in controls; χ^2^ *p* = 0.0525). However, sexual activity within the prior six months differed substantially (χ^2^ *p* < 0.0001): 59.4% of the participants on haemodialysis were sexually active versus 93.2% of participants with CKD and 75.9% of controls, with significant post hoc differences for haemodialysis versus CKD (Bonferroni *p* < 0.0001) and CKD versus controls (Bonferroni *p* = 0.0072).

Anxiety/fear/insomnia was analysed as self-reported symptom presence at enrolment, using the binary categories displayed in Table 1. Symptom presence was reported by 100.0% (96/96) of the haemodialysis group, compared with 33.0% (29/88) of the CKD group and 19.3% (16/83) of the controls (χ^2^ *p* < 0.0001). Bonferroni-adjusted post hoc comparisons indicated a higher symptom presence in the haemodialysis versus the CKD group (*p* < 0.0001) and in the haemodialysis group versus controls (*p* < 0.0001).

### 3.2. Mixed Effects Models of Sexual Function

We analysed the IIEF-15 total score and its five domains using linear mixed effects models with a random intercept to account for repeated annual measurements within individuals. Fixed effects included year, group, age, EQ-5D-5L and diabetes status (type 1, type 2; and reference: no diabetes). Table 2 presents adjusted fixed effect estimates (β [95% CI]), where negative values indicate lower (worse) scores relative to the reference category.

**Figure 1:**
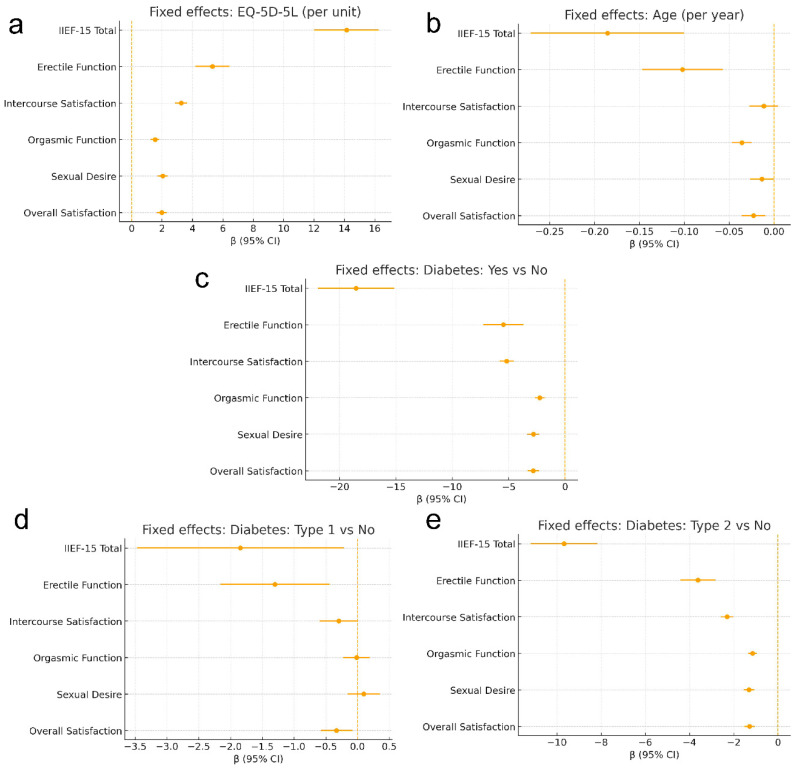
Fixed effect coefficients for key covariates across IIEF-15 total score and subdomains in men with non-dialysis CKD, receiving haemodialysis and in the control participants. Notes: Each panel shows a forest plot of the adjusted regression coefficient (β) with its 95% confidence interval (CI) for one covariate. EQ-5D-5L index score per unit (**a**) and age per year (**b**) are estimated from the primary mixed effects models. Diabetes effects are shown under two alternative specifications: a binary indicator (Diabetes: yes vs. no) in panel (**c**) from sensitivity models, and diabetes type contrasts (type 1 vs. no diabetes; type 2 vs. no diabetes) in panels (**d**,**e**) from the primary models. The vertical dashed line at β = 0 indicates no association. Negative β values indicate lower (worse) IIEF-15 scores, whereas positive β values indicate higher scores associated with better health-related quality of life. Models use repeated measures over three annual visits and include a random intercept to account for within-participant correlation.

In relation to the primary outcome, longitudinal erectile function scores declined over follow-up. Compared with Year 1, erectile function was lower at Year 2 (β = −0.723; *p* = 0.028) and further reduced at Year 3 (β = −1.048; *p* = 0.0015). Group contrasts indicated lower erectile function in the CKD group compared with controls (β = −0.974; *p* = 0.0026), while the haemodialysis contrast did not reach conventional statistical significance (β = −1.881; *p* = 0.063) (Figure 1).

**Figure 2:**
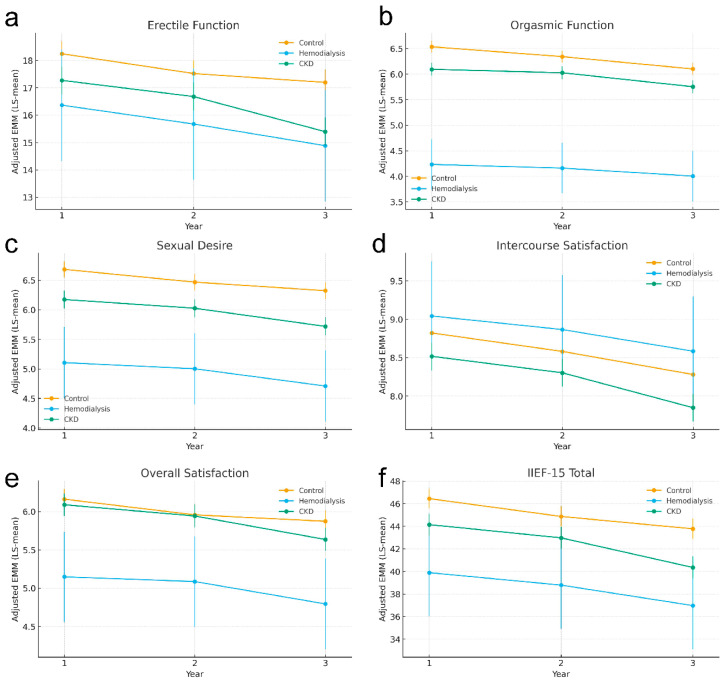
Adjusted trajectories of IIEF-15 total score and subdomains over three years in the control, and the CKD and haemodialysis groups. Notes: Panels depict estimated marginal means (LS-means) of the IIEF-15 total score (**f**) and each subscale (erectile function (**a**), orgasmic function (**b**), sexual desire (**c**), intercourse satisfaction (**d**), and overall satisfaction (**e**)) at Years 1, 2 and 3 for participants with chronic kidney disease (CKD), patients on haemodialysis, and control participants without CKD. Lines connect LS means over time for each group, with circles indicating the point estimates and vertical bars representing 95% confidence intervals derived from linear mixed effects models. The *x*-axis shows the study year (1–3), and the *y*-axis shows the adjusted IIEF-15 score in the respective domain. Estimates are adjusted for relevant covariates (including age, health-related quality of life, and diabetes status), with random effects accounting for repeated observations within individuals. Higher values indicate better erectile function, sexual function and satisfaction.

For the secondary outcomes, the IIEF-15 total score showed a clear deterioration over time (Year 2 vs. Year 1: β = −1.578 with *p* = 0.011; Year 3 vs. Year 1: β = −2.675 with *p* < 0.001), alongside persistent between-group differences. Relative to the controls, total scores were lower in the CKD group (β = −2.303; *p* = 0.0002) and substantially lower in the haemodialysis group (β = −6.557; *p* = 0.0007). Similar time-related declines were observed across the remaining domains, including intercourse satisfaction, orgasmic function, sexual desire and overall satisfaction (all *p* ≤ 0.036 at Year 2 and all *p* ≤ 0.0025 at Year 3). Compared with controls, the patients with CKD scored lower for intercourse satisfaction (β = −0.305; *p* = 0.007), orgasmic function (β = −0.441; *p* < 0.001) and sexual desire (β = −0.511; *p* < 0.001), whereas overall satisfaction did not differ (β = −0.072; *p* = 0.440). In the haemodialysis group, the largest deficits were seen for orgasmic function (β = −2.302; *p* < 0.001), sexual desire (β = −1.580; *p* < 0.001) and overall satisfaction (β = −1.015; *p* = 0.0006), while intercourse satisfaction did not differ from controls (β = +0.221; *p* = 0.533) (Figure 2).

Across both primary and secondary analyses, covariate patterns were consistent and clinically interpretable. Being of an older age was independently associated with a lower IIEF-15 total and most domain scores (e.g., total: β = −0.186 per year; erectile function: β = −0.102 per year; both *p* < 0.001), with no clear association for intercourse satisfaction (*p* = 0.157). Having a higher health-related quality of life (EQ-5D-5L) was strongly and uniformly associated with better sexual function across all outcomes (all *p* < 0.001). Diabetes, particularly type 2, was consistently associated with worse sexual function across total and all domains (all *p* < 0.001), whereas type 1 diabetes showed smaller and less consistent associations, most notably for total score, erectile function and overall satisfaction. Sensitivity analyses using a binary diabetes indicator (yes/no) yielded directionally consistent results with the type-specific specification, confirming diabetes as an independent adverse correlate of IIEF outcomes across domains. The principal clinical interpretation was unchanged: type 2 diabetes showed the largest and most consistent deficits in the primary (type-specific) models, while the binary specification provided a summary estimate of the overall diabetes burden. Sensitivity analyses are reported in the Supplementary Tables (Tables S1–S12), together with the full set of group-by-year interaction terms.

## 4. Discussion

In this three-year prospective study, men with CKD exhibited persistent between-group stratification in sexual function, with the haemodialysis group consistently the lowest, the non-dialysis stage 3 CKD group the intermediate, and the controls the highest. Mixed effects models indicated modest but statistically robust declines over time in several domains, while the non-significant group × year interaction supports broadly parallel longitudinal change without evidence of convergence or divergence between groups. The non-significant group × year interaction indicates that between-group differences were already evident at the first assessment and then remained broadly parallel over follow-up, consistent with an early-established burden that does not rapidly converge or diverge over three years. Across outcomes, lower health-related quality of life, older age and diabetes—particularly type 2—were independently associated with lower IIEF-15 scores. These longitudinal findings extend prior syntheses that emphasise the heavy burden of ED across CKD and dialysis populations by adding temporal resolution beyond cross-sectional estimates [12,23,30,31].

Beyond statistical significance, the observed pattern has direct clinical implications because sexual dysfunction in CKD is not an isolated quality-of-life concern but a multidimensional outcome that integrates vascular health, metabolic comorbidity, endocrine milieu, symptom burden and psychosocial context [2,15,17]. Importantly, baseline group comparability in age, alongside clear differences in BMI and sociodemographic composition (education, employment and urban/rural residence), underscores that the sexual-function gradient likely reflects both biomedical disease severity and the lived context of CKD and dialysis care [9,12]. Equally, the relationship and behavioural context captured at enrolment, and the high prevalence of reported sexual partnership but the markedly lower recent sexual activity in the haemodialysis group, provide a clinically meaningful backdrop for interpreting IIEF-15 trajectories: it suggests that reduced the sexual function in the haemodialysis group is accompanied with reduced sexual engagement, which may further compound dysfunction through relational strain, reduced confidence and avoidance behaviours [9,32]. The consistent positive association between EQ-5D-5L and IIEF outcomes reinforces the interpretation that sexual function in this population is tightly coupled with overall health status and symptom experience, rather than reflecting a narrowly genital or purely psychogenic phenomenon [1,32,33]. Consequently, the stability of between-group separation over time argues for early, structured assessment and management of sexual dysfunction from the point of CKD recognition and at dialysis initiation, with attention to potentially modifiable drivers (notably glycaemic control, symptom burden and quality-of-life determinants), rather than viewing ED as an inevitable late complication that can be deferred [14,15,22].

Placing our results in context, reported ED prevalence commonly ranges from approximately one-half to three-quarters among men with CKD or on dialysis, with greater severity in the presence of diabetes and hypertension [23]. The independent associations we observed for age and diabetes, together with the strong positive relationship between EQ-5D-5L and IIEF outcomes, are consistent with these established correlates [1,9,33]. In line with reports that ED in haemodialysis often approaches or exceeds two-thirds prevalence, the haemodialysis group remained lowest at each time point, underscoring that dialysis does not normalise sexual health [9,34]. Comparative work has suggested more favourable erectile outcomes with continuous ambulatory peritoneal dialysis relative to haemodialysis and has highlighted the adverse role of anxiety symptoms [32,35]. Although our cohort did not include a CAPD stratum, the persistently lower scores in haemodialysis are coherent with a modality gradient and with the psychosocial dimension described elsewhere; the positive association between EQ-5D-5L and IIEF supports this interpretation [32].

Several CKD-related factors may contribute to poorer sexual function, including vascular disease, metabolic comorbidity and endocrine disturbance, particularly in dialysis populations [36,37]. While we did not systematically measure arterial stiffness indices or sex hormones, the stable between-group separation alongside consistent age and diabetes effects is compatible with a multifactorial burden that is already present at study entry and then changes only modestly over time. Evidence from transplant populations generally indicates improvement relative to dialysis, albeit with variable recovery and the age-dependent persistence of ED [31,38,39,40]. Although transplant recipients were excluded here, the durability of the haemodialysis deficit is consistent with the view that the restoration of kidney function—rather than dialysis adequacy alone—is often required for substantial recovery.

From a clinical perspective, therapeutic signals within CKD and dialysis cohorts reinforce the implications of these findings. Adjusted-dose tadalafil appears to improve IIEF domains in haemodialysis, intracavernosal alprostadil can yield substantial gains in selected series, and sildenafil responsiveness may be enhanced post transplant [41,42,43]. Against this background, our results support the earlier identification of ED in CKD, systematic assessment of quality-of-life determinants, and proactive optimisation of comorbid diabetes, alongside the thoughtful use of renal-appropriate ED therapies rather than deferring intervention until sexual dysfunction is advanced and entrenched. Accordingly, these findings generalise most directly to men with stage 3 CKD and on haemodialysis in similar clinical settings and should not be extrapolated to non-dialysis stages 4–5 without corroborating data.

Taken together, the data indicate a high burden of sexual dysfunction that scales with CKD severity, modest declines over three years, and consistent adverse associations with age and diabetes, particularly type 2. The absence of differential decline across groups supports embedding ED assessment and management into CKD care pathways from early stages and at dialysis initiation, with particular attention to men with type 2 diabetes and impaired health-related quality of life [23,31,32,34,35,36,37,38,39,40,41,42,43].

## 5. Conclusions

The strengths of this study include the prospective three-year design with repeated measures and the use of mixed effects models that account for within-person correlation while providing adjusted estimates for key clinical covariates. This framework allows clear visualisation of between-group separation over time and yields interpretable estimates for the influence of age, diabetes status and health-related quality of life. The use of the full IIEF-15, capturing multiple domains of sexual function, alongside standardised assessment of health-related quality of life, further enhances clinical interpretability.

The limitations of the study include its restriction to stage 3 CKD in the non-dialysis cohort and the absence of transplantation or CAPD strata, which precludes direct comparison across additional renal replacement modalities. Data on biological biomarkers, such as sex hormones or arterial stiffness indices, were not systematically collected, limiting causal attribution to specific vascular or endocrine pathways. Residual confounding by unmeasured psychosocial factors, medication exposures, or dialysis adequacy remains possible, and mixed effects models rely on the assumption that missing data are missing at random. Notably, the very high prevalence of self-reported anxiety/fear/insomnia symptoms in haemodialysis participants suggests a substantial psychological and sleep-related burden that could confound or mediate sexual outcomes; the absence of comprehensive mental health assessment (e.g., validated depression/anxiety scales) limits causal attribution. Because the cohort was predominantly older and recruited from specific clinical/community settings, generalisability of the findings to younger men with CKD may be limited and should be interpreted cautiously. These considerations warrant cautious interpretation of causal explanations; however, they do not alter the central longitudinal finding of persistent, graded impairment, with the haemodialysis group exhibiting the lowest score at all time points.

Male sexual function is impaired in a graded manner across the CKD spectrum, with haemodialysis patients consistently scoring lower than the non-dialysis CKD group and controls, and with no evidence of divergent trajectories between groups over a three-year period. A lower EQ-5D-5L, older age and, in particular, type 2 diabetes independently predict poorer IIEF-15 outcomes. Embedding management strategies that include early identification of ED, systematic attention to quality of life, optimisation of metabolic comorbidity and appropriate ED treatment within CKD care pathways should be evaluated in future trials in both dialysis and pre-dialysis populations.

## Figures and Tables

**Table 1:** Demographic and baseline characteristics by group.

Variable	Haemodialysis	CKD (eGFR < 60)	Control Group	*p*-Value (Omnibus)	Significant Bonferroni-Adjusted Pairs
**Age**	63.00 [53.00, 70.00]	67.50 [57.75, 72.00]	65.00 [55.00, 70.50]	0.1104 (KW)	
**BMI**	24.85 [21.67, 27.84]	27.09 [25.50, 29.62]	26.60 [24.20, 28.85]	<0.0001 (KW)	Haemodialysis–CKD = <0.0001; Haemodialysis–Control = 0.0211
**Education level**	Tertiary: 59 (61.5%)	Secondary: 49 (55.7%)	Tertiary: 52 (62.7%)	0.0106 (Χ^2^)	Haemodialysis–CKD = 0.0130; CKD–Control = 0.0360
**Nationality**	Greek: 89 (92.7%)	Greek: 87 (98.9%)	Greek: 83 (100.0%)	0.0337 (Χ^2^)	
**Marital status**	Married: 69 (71.9%)	Married: 72 (81.8%)	Married: 46 (55.4%)	0.0051 (Χ^2^)	CKD–Control = 0.0085
**Children**	Yes: 76 (79.2%)	Yes: 70 (79.5%)	Yes: 59 (71.1%)	0.3338 (Χ^2^)	
**Employment status**	Retired: 75 (78.1%)	Retired: 57 (64.8%)	Employed: 40 (48.2%)	<0.0001 (Χ^2^)	Haemodialysis–CKD = <0.0001; Haemodialysis–Control = <0.0001; CKD–Control = 0.0455
**Smoking**	Yes: 44 (45.8%) No 30 (31.3%) Former 22 (22.9%)	Yes 28 (31.8%) No 21 (23.9%) Former 39 (44.3%)	Yes: 32 (38.6%) No 27 (32.5%) Former 24 (28.9%)	0.0320 (Χ^2^)	Haemodialysis–CKD = 0.0253
**Residence (urban/rural)**	Urban: 54 (56.2%)	Urban: 75 (85.2%)	Urban: 66 (79.5%)	<0.0001 (Χ^2^)	Haemodialysis–CKD = <0.0001; Haemodialysis–Control = 0.0040
**Diabetes mellitus status**	No: 74 (77.1%)	No: 56 (63.6%)	No: 66 (79.5%)	0.0793 (Χ^2^)	
**Monogamous/Polygamous**	Monogamous: 85 (88.5%)	Monogamous: 87 (98.9%)	Monogamous: 70 (84.3%)	0.0006 (Χ^2^)	Haemodialysis–CKD = 0.0499; CKD–Control = 0.0014
**Having a sexual partner**	Yes: 82 (85.4%)	Yes: 84 (95.5%)	Yes: 71 (85.5%)	0.0525 (Χ^2^)	
**Sexually active**	Yes: 57 (59.4%)	Yes: 82 (93.2%)	Yes: 63 (75.9%)	<0.0001 (Χ^2^)	Haemodialysis–CKD = <0.0001; CKD–Control = 0.0072
**Anxiety/Fear/Insomnia (presence)**	Yes: 96 (100.0%)	No: 59 (67.0%)	No: 67 (80.7%)	<0.0001 (Χ^2^)	Haemodialysis–CKD = <0.0001; Haemodialysis–Control = <0.0001

Categorical cells show the modal category per group with count and percent. Pairwise *p*-values are Bonferroni adjusted and only significant pairs are listed. KW = Kruskal–Wallis., X^2^ Chi squared test.

**Table 2:** Fixed effects estimates (β [95% CI], *p*) for IIEF-15 total and subscales scores.

Predictor (Ref)	IIEF-15 Total	Erectile Function	Intercourse Satisfaction	Orgasmic Function	Sexual Desire	Overall Satisfaction
**Year 2 vs. Year 1**	−1.578 [−2.798, −0.358], *p* = 0.011	−0.723 [−1.367, −0.079], *p* = 0.028	−0.241 [−0.466, −0.016], *p* = 0.036	−0.193 [−0.349, −0.037], *p* = 0.016	−0.217 [−0.407, −0.027], *p* = 0.026	−0.205 [−0.392, −0.018], *p* = 0.032
**Year 3 vs. Year 1**	−2.675 [−3.895, −1.455], *p* < 0.001	−1.048 [−1.692, −0.404], *p* = 0.0015	−0.542 [−0.768, −0.317], *p* < 0.001	−0.434 [−0.590, −0.278], *p* < 0.001	−0.361 [−0.552, −0.171], *p* = 0.0002	−0.289 [−0.476, −0.102], *p* = 0.0025
**CKD vs. Control**	−2.303 [−3.501, −1.105], *p* = 0.0002	−0.974 [−1.606, −0.341], *p* = 0.0026	−0.305 [−0.526, −0.083], *p* = 0.007	−0.441 [−0.595, −0.288], *p* < 0.001	−0.511 [−0.698, −0.324], *p* < 0.001	−0.072 [−0.256, 0.111], *p* = 0.440
**Hemodialysis vs. Control**	−6.557 [−10.319, −2.796], *p* = 0.0007	−1.881 [−3.866, 0.105], *p* = 0.063	+0.221 [−0.474, 0.915], *p* = 0.533	−2.302 [−2.784, −1.821], *p* < 0.001	−1.580 [−2.167, −0.993], *p* < 0.001	−1.015 [−1.591, −0.438], *p* = 0.0006
**Age (per year)**	−0.186 [−0.271, −0.100], *p* < 0.001	−0.102 [−0.147, −0.057], *p* < 0.001	−0.011 [−0.027, 0.004], *p* = 0.157	−0.036 [−0.047, −0.025], *p* < 0.001	−0.014 [−0.027, −0.000], *p* = 0.047	−0.023 [−0.036, −0.010], *p* = 0.0007
**EQ-5D-5L (per unit)**	+14.145 [+12.020, +16.270], *p* < 0.001	+5.317 [+4.195, +6.439], *p* < 0.001	+3.257 [+2.865, +3.650], *p* < 0.001	+1.545 [+1.273, +1.817], *p* < 0.001	+2.044 [+1.713, +2.376], *p* < 0.001	+1.982 [+1.656, +2.307], *p* < 0.001
**Diabetes: Type 1 vs. No**	−1.845 [−3.472, −0.219], *p* = 0.026	−1.300 [−2.159, −0.442], *p* = 0.003	−0.297 [−0.597, 0.004], *p* = 0.053	−0.015 [−0.223, 0.193], *p* = 0.887	+0.097 [−0.157, 0.351], *p* = 0.452	−0.330 [−0.580, −0.081], *p* = 0.0096
**Diabetes: Type 2 vs. No**	−9.675 [−11.184, −8.166], *p* < 0.001	−3.621 [−4.418, −2.825], *p* < 0.001	−2.306 [−2.585, −2.028], *p* < 0.001	−1.151 [−1.344, −0.958], *p* < 0.001	−1.311 [−1.546, −1.075], *p* < 0.001	−1.286 [−1.517, −1.054], *p* < 0.001
**Diabetes: Yes vs. No**	−18.529 [−21.915, −15.143], *p* < 0.001	−5.468 [−7.255, −3.682], *p* < 0.001	−5.181 [−5.806, −4.556], *p* < 0.001	−2.248 [−2.681, −1.814], *p* < 0.001	−2.813 [−3.341, −2.285], *p* < 0.001	−2.819 [−3.338, −2.300], *p* < 0.001

## Data Availability

The datasets generated and/or analysed during the current study are not publicly available due to privacy and ethical restrictions, as they contain personal and sensitive health information that could compromise participant confidentiality. De-identified data may be made available from the corresponding author upon reasonable request and subject to approval by the relevant Ethics Committees/Institutional Review Boards and completion of an appropriate data-sharing agreement, in accordance with applicable data protection regulations.

## Supplementary Materials

The following supporting information can be downloaded at https://www.mdpi.com/article/10.3390/jcm15041402/s1. Table S1. Coefficient estimates for “IIEF-15 Total Score”. Reference: Year 1 and Control group. Table S2. EMMs (LS-means) for “IIEF-15 Total Score” by Year × Group (adjusted at mean Age/EQ-5D-5L; modal Diabetes). Table S3. Coefficient estimates for “Erectile Function”. Reference: Year 1 and Control group. Table S4. EMMs (LS-means) for “Erectile Function” by Year × Group (adjusted at mean Age/EQ-5D-5L; modal Diabetes). Table S5. Coefficient estimates for “Intercourse Satisfaction”. Reference: Year 1 and Control group. Table S6. EMMs (LS-means) for “Intercourse Satisfaction” by Year × Group (adjusted at mean Age/EQ-5D-5L; modal Diabetes). Table S7. Coefficient estimates for “Orgasmic Function”. Reference: Year 1 and Control group. Table S8. EMMs (LS-means) for “Orgasmic Function” by Year × Group (adjusted at mean Age/EQ-5D-5L; modal Diabetes). Table S9. Coefficient estimates for “Sexual Desire”. Reference: Year 1 and Control group. Table S10. EMMs (LS-means) for “Sexual Desire” by Year × Group (adjusted at mean Age/EQ-5D-5L; modal Diabetes). Table S11. Coefficient estimates for “Overall Satisfaction”. Reference: Year 1 and Control group. Table S12. EMMs (LS-means) for “Overall Satisfaction” by Year × Group (adjusted at mean Age/EQ-5D-5L; modal Diabetes).

## Abbreviations

The following abbreviations are used in this manuscript:

ANOVA	Analysis of variance
BMI	Body mass index
CAPD	Continuous ambulatory peritoneal dialysis
CI	Confidence interval
CKD	Chronic kidney disease
CKD-EPI	Chronic kidney disease epidemiology collaboration (equation)
ECM	Extracellular matrix
ED	Erectile dysfunction
EF	Erectile function (IIEF domain)
eGFR	Estimated glomerular filtration rate
EMMs	Estimated marginal means
EQ-5D-5L	EuroQol 5-dimension, 5-level health-related quality-of-life instrument
ESRD	End-stage renal disease
FSH	Follicle-stimulating hormone
IIEF-15	International Index of Erectile Function (15-item version)
IIEF-5	International Index of Erectile Function (5-item version)
IQR	Interquartile range
KDIGO	Kidney disease: improving global outcomes
KW	Kruskal–Wallis (test)
LH	Luteinising hormone
LS-means	Least-squares means
*p*	*p*-value
SD	Standard deviation
β	Regression coefficient
χ2	Chi-squared (test)
