# Supplementary material for: Erectile Function Decline in Men with Chronic Kidney Disease: A Three-Year Case–Control Study Comparing Haemodialysis, Non-Dialysis CKD and Community Controls
Source: J Clin Med. 2026 Feb 11;15(4):1402. doi: 10.3390/jcm15041402 (PMC12942453; doi:10.3390/jcm15041402)
Supplement: Supplementary file 1 [file jcm-15-01402-s001.zip › jcm-4138516-supplementary.pdf]

IIEF-15 Total Score

Table S1 . Coefficient estimates for "IIEF-15 Total Score". Reference: Year 1 and Control group.

Term	β	SE	t	p	95% CI low	95% CI high
Intercept	52.2414	3.0329	17.225	0.0000	46.2834	58.1995
C(Year)[T.2]	-1.5783	0.6210	-2.541	0.0113	-2.7983	-0.3583
C(Year)[T.3]	-2.6747	0.6210	-4.307	0.0000	-3.8947	-1.4547
C(Group)[T.Hemodialysis]	-6.5571	1.9147	-3.425	0.0007	-10.3186	-2.7956
C(Group)[T.CKD]	-2.3027	0.6099	-3.775	0.0002	-3.5009	-1.1046
**Sensitivity analysis** C(Diabetes)[T.Yes]	-18.5288	1.7235	-10.751	0.0000	-21.9145	-15.1431
C(Diabetes)[T.Type 1]	-1.8454	0.8281	-2.228	0.0263	-3.4723	-0.2186
C(Diabetes)[T.Type 2]	-9.6749	0.7681	-12.596	0.0000	-11.1837	-8.1660
C(Year)[T.2] : C(Group)[T.Hemodialysis]	0.4741	0.8480	0.559	0.5763	-1.1918	2.1401
C(Year)[T.3] : C(Group)[T.Hemodialysis]	-0.2420	0.8480	-0.285	0.7755	-1.9079	1.4240
C(Year)[T.2] : C(Group)[T.CKD]	0.4079	0.8657	0.471	0.6377	-1.2928	2.1085
C(Year)[T.3] : C(Group)[T.CKD]	-1.1208	0.8657	-1.295	0.1960	-2.8214	0.5799
Age	-0.1855	0.0435	-4.264	0.0000	-0.2710	-0.1000
EQ-5D-5L	14.1449	1.0819	13.075	0.0000	12.0196	16.2702

Table S2. EMMs (LS-means) for "IIEF-15 Total Score" by Year×Group (adjusted at mean Age/EQ-5D-5L; modal Diabetes).

Year	Group	LS-Mean	SE	95% CI low	95% CI high
1	Control	46.448	0.442	45.582	47.313
2	Control	44.869	0.459	43.970	45.768
3	Control	43.773	0.459	42.874	44.672
1	Hemodialysis	39.890	1.968	36.034	43.747
2	Hemodialysis	38.786	1.968	34.930	42.643
3	Hemodialysis	36.974	1.968	33.117	40.831
1	CKD	44.145	0.492	43.181	45.109
2	CKD	42.974	0.497	42.001	43.948
3	CKD	40.349	0.497	39.376	41.323

Erectile Function

Table S3. Coefficient estimates for "Erectile Function". Reference: Year 1 and Control group.

Term	β	SE	t	p	95% CI low	95% CI high
Intercept	22.4221	1.6007	14.008	0.0000	19.2776	25.5665
C(Year)[T.2]	-0.7229	0.3278	-2.206	0.0278	-1.3668	-0.0790
C(Year)[T.3]	-1.0482	0.3278	-3.198	0.0015	-1.6921	-0.4043
C(Group)[T.Hemodialysis]	-1.8807	1.0105	-1.861	0.0633	-3.8658	0.1045
C(Group)[T.CKD]	-0.9735	0.3219	-3.024	0.0026	-1.6058	-0.3411
**Sensitivity analysis** C(Diabetes)[T.Yes]	-5.4683	0.9096	-6.012	0.0000	-7.2551	-3.6815

C(Diabetes)[T.Type 1]	-1.3003	0.4371	-2.975	0.0031	-2.1588	-0.4417
C(Diabetes)[T.Type 2]	-3.6214	0.4054	-8.934	0.0000	-4.4177	-2.8251
C(Year)[T.2] : C(Group)[T.Hemodialysis]	0.0354	0.4476	0.079	0.9370	-0.8438	0.9146
C(Year)[T.3] : C(Group)[T.Hemodialysis]	-0.4310	0.4476	-0.963	0.3360	-1.3102	0.4482
C(Year)[T.2] : C(Group)[T.CKD]	0.1320	0.4569	0.289	0.7728	-0.7656	1.0295
C(Year)[T.3] : C(Group)[T.CKD]	-0.8268	0.4569	-1.810	0.0709	-1.7244	0.0707
Age	-0.1020	0.0230	-4.442	0.0000	-0.1471	-0.0569
EQ-5D-5L	5.3169	0.5710	9.312	0.0000	4.1952	6.4385

Table S4. EMMs (LS-means) for “Erectile Function” by Year×Group (adjusted at mean Age/EQ-5D-5L; modal Diabetes).

Year	Group	LS-Mean	SE	95% CI low	95% CI high
1	Control	18.243	0.233	17.786	18.700
2	Control	17.520	0.242	17.046	17.995
3	Control	17.195	0.242	16.721	17.670
1	Hemodialysis	16.363	1.038	14.327	18.398
2	Hemodialysis	15.675	1.038	13.640	17.711
3	Hemodialysis	14.884	1.038	12.848	16.919
1	CKD	17.270	0.260	16.761	17.779
2	CKD	16.679	0.262	16.165	17.193
3	CKD	15.395	0.262	14.881	15.908

Intercourse Satisfaction

Table S5. Coefficient estimates for “Intercourse Satisfaction”. Reference: Year 1 and Control group.

Term	β	SE	t	p	95% CI low	95% CI high
EQ-5D-5L	3.2571	0.1998	16.303	0.0000	2.8646	3.6496
**Sensitivity analysis** C(Diabetes)[T.Yes]	-5.1809	0.3183	-16.278	0.0000	-5.8061	-4.5556
C(Diabetes)[T.Type 2]	-2.3064	0.1418	-16.261	0.0000	-2.5851	-2.0278
C(Year)[T.3]	-0.5422	0.1147	-4.727	0.0000	-0.7675	-0.3169
C(Group)[T.CKD]	-0.3047	0.1126	-2.705	0.0071	-0.5259	-0.0834
C(Year)[T.2]	-0.2410	0.1147	-2.101	0.0361	-0.4663	-0.0157
C(Diabetes)[T.Type 1]	-0.2970	0.1529	-1.942	0.0527	-0.5974	0.0035
Age	-0.0114	0.0080	-1.416	0.1573	-0.0272	0.0044
C(Year)[T.3] : C(Group)[T.CKD]	-0.1283	0.1599	-0.802	0.4227	-0.4423	0.1858
C(Group)[T.Hemodialysis]	0.2205	0.3536	0.623	0.5332	-0.4742	0.9151
C(Year)[T.3] : C(Group)[T.Hemodialysis]	0.0838	0.1566	0.535	0.5926	-0.2238	0.3915
C(Year)[T.2] : C(Group)[T.Hemodialysis]	0.0639	0.1566	0.408	0.6835	-0.2438	0.3715
C(Year)[T.2] : C(Group)[T.CKD]	0.0251	0.1599	0.157	0.8755	-0.2890	0.3391
Intercept	8.2131	0.5601	14.664	0.0000	7.1129	9.3134

Table S6. EMMs (LS-means) for “Intercourse Satisfaction” by Year×Group (adjusted at mean Age/EQ-5D-5L; modal Diabetes).

Year	Group	LS-Mean	SE	95% CI low	95% CI high
1	Control	8.822	0.082	8.662	8.982
2	Control	8.581	0.085	8.415	8.747
3	Control	8.280	0.085	8.114	8.446
1	Hemodialysis	9.043	0.363	8.330	9.755
2	Hemodialysis	8.866	0.363	8.153	9.578
3	Hemodialysis	8.584	0.363	7.872	9.297
1	CKD	8.517	0.091	8.339	8.696
2	CKD	8.302	0.092	8.122	8.481
3	CKD	7.847	0.092	7.667	8.027

Orgasmic Function

Table S7. Coefficient estimates for “Orgasmic Function”. Reference: Year 1 and Control group.

Term	β	SE	t	p	95% CI low	95% CI high
C(Diabetes)[T.Type 2]	-1.1508	0.0983	-11.705	0.0000	-1.3439	-0.9577
EQ-5D-5L	1.5453	0.1385	11.159	0.0000	1.2732	1.8173
**Sensitivity analysis** C(Diabetes)[T.Yes]	-2.2476	0.2206	-10.188	0.0000	-2.6810	-1.8143
C(Group)[T.Hemodialysis]	-2.3021	0.2451	-9.393	0.0000	-2.7835	-1.8206
Age	-0.0358	0.0056	-6.429	0.0000	-0.0467	-0.0249
C(Group)[T.CKD]	-0.4411	0.0781	-5.650	0.0000	-0.5945	-0.2878
C(Year)[T.3]	-0.4337	0.0795	-5.456	0.0000	-0.5899	-0.2776
C(Year)[T.2]	-0.1928	0.0795	-2.425	0.0156	-0.3489	-0.0366
C(Year)[T.3] : C(Group)[T.Hemodialysis]	0.2046	0.1085	1.885	0.0600	-0.0087	0.4178
C(Year)[T.2] : C(Group)[T.CKD]	0.1246	0.1108	1.124	0.2614	-0.0931	0.3423
C(Year)[T.2] : C(Group)[T.Hemodialysis]	0.1199	0.1085	1.104	0.2700	-0.0934	0.3331
C(Year)[T.3] : C(Group)[T.CKD]	0.0928	0.1108	0.838	0.4026	-0.1249	0.3105
C(Diabetes)[T.Type 1]	-0.0151	0.1060	-0.143	0.8865	-0.2234	0.1931
Intercept	8.1318	0.3882	20.947	0.0000	7.3692	8.8945

Table S8. EMMs (LS-means) for "Orgasmic Function" by Year×Group (adjusted at mean Age/EQ-5D-5L; modal Diabetes).

Year	Group	LS-Mean	SE	95% CI low	95% CI high
1	Control	6.535	0.057	6.424	6.646
2	Control	6.342	0.059	6.227	6.457
3	Control	6.101	0.059	5.986	6.216
1	Hemodialysis	4.233	0.252	3.739	4.727
2	Hemodialysis	4.160	0.252	3.666	4.654
3	Hemodialysis	4.004	0.252	3.510	4.498
1	CKD	6.094	0.063	5.971	6.217
2	CKD	6.026	0.064	5.901	6.150
3	CKD	5.753	0.064	5.629	5.878

Sexual Desire

Table S9. Coefficient estimates for "Sexual Desire". Reference: Year 1 and Control group.

Term	β	SE	t	p	95% CI low	95% CI high
EQ-5D-5L	2.0441	0.1688	12.113	0.0000	1.7126	2.3756
C(Diabetes)[T.Type 2]	-1.3106	0.1198	-10.939	0.0000	-1.5460	-1.0753
**Sensitivity analysis** C(Diabetes)[T.Yes]	-2.8129	0.2688	-10.463	0.0000	-3.3410	-2.2848
C(Group)[T.CKD]	-0.5111	0.0951	-5.372	0.0000	-0.6980	-0.3242
C(Group)[T.Hemodialysis]	-1.5801	0.2987	-5.291	0.0000	-2.1669	-0.9934
C(Year)[T.3]	-0.3614	0.0969	-3.731	0.0002	-0.5517	-0.1711
C(Year)[T.2]	-0.2169	0.0969	-2.239	0.0256	-0.4072	-0.0266
Age	-0.0135	0.0068	-1.990	0.0471	-0.0268	-0.0002
C(Year)[T.2] : C(Group)[T.Hemodialysis]	0.1127	0.1323	0.852	0.3946	-0.1472	0.3726
C(Diabetes)[T.Type 1]	0.0972	0.1292	0.752	0.4522	-0.1566	0.3509
C(Year)[T.3] : C(Group)[T.CKD]	-0.0931	0.1350	-0.689	0.4909	-0.3584	0.1722
C(Year)[T.2] : C(Group)[T.CKD]	0.0691	0.1350	0.512	0.6089	-0.1961	0.3344
C(Year)[T.3] : C(Group)[T.Hemodialysis]	-0.0344	0.1323	-0.260	0.7950	-0.2942	0.2255
Intercept	6.6977	0.4731	14.158	0.0000	5.7684	7.6271

Table S10. EMMs (LS-means) for “Sexual Desire” by Year×Group (adjusted at mean Age/EQ-5D-5L; modal Diabetes).

Year	Group	LS-Mean	SE	95% CI low	95% CI high
1	Control	6.685	0.069	6.550	6.820
2	Control	6.468	0.072	6.328	6.609
3	Control	6.324	0.072	6.183	6.464
1	Hemodialysis	5.105	0.307	4.503	5.707
2	Hemodialysis	5.001	0.307	4.399	5.602
3	Hemodialysis	4.709	0.307	4.108	5.311
1	CKD	6.174	0.077	6.024	6.324
2	CKD	6.026	0.077	5.874	6.178
3	CKD	5.719	0.077	5.568	5.871

Overall Satisfaction

Table S11. Coefficient estimates for “Overall Satisfaction”. Reference: Year 1 and Control group.

Term	β	SE	t	p	95% CI low	95% CI high
EQ-5D-5L	1.9816	0.1659	11.946	0.0000	1.6557	2.3074
C(Diabetes)[T.Type 2]	-1.2856	0.1178	-10.917	0.0000	-1.5170	-1.0543
**Sensitivity analysis** C(Diabetes)[T.Yes]	-2.8191	0.2642	-10.668	0.0000	-3.3382	-2.3000
C(Group)[T.Hemodialysis]	-1.0147	0.2936	-3.456	0.0006	-1.5914	-0.4380
Age	-0.0228	0.0067	-3.420	0.0007	-0.0359	-0.0097
C(Year)[T.3]	-0.2892	0.0952	-3.037	0.0025	-0.4762	-0.1021
C(Diabetes)[T.Type 1]	-0.3302	0.1270	-2.601	0.0096	-0.5797	-0.0808
C(Year)[T.2]	-0.2048	0.0952	-2.151	0.0319	-0.3919	-0.0178
C(Year)[T.3] : C(Group)[T.CKD]	-0.1654	0.1327	-1.246	0.2133	-0.4261	0.0954
C(Year)[T.2] : C(Group)[T.Hemodialysis]	0.1423	0.1300	1.095	0.2742	-0.1131	0.3977
C(Group)[T.CKD]	-0.0723	0.0935	-0.773	0.4396	-0.2560	0.1114
C(Year)[T.3] : C(Group)[T.Hemodialysis]	-0.0650	0.1300	-0.500	0.6173	-0.3204	0.1904
C(Year)[T.2] : C(Group)[T.CKD]	0.0571	0.1327	0.430	0.6673	-0.2037	0.3178
Intercept	6.7767	0.4650	14.573	0.0000	5.8631	7.6902

Table S12. EMMs (LS-means) for “Overall Satisfaction” by Year×Group (adjusted at mean Age/EQ-5D-5L; modal Diabetes).

Year	Group	LS-Mean	SE	95% CI low	95% CI high
1	Control	6.162	0.068	6.029	6.295
2	Control	5.957	0.070	5.819	6.095
3	Control	5.873	0.070	5.735	6.010
1	Hemodialysis	5.147	0.302	4.556	5.738
2	Hemodialysis	5.085	0.302	4.493	5.676
3	Hemodialysis	4.793	0.302	4.202	5.384
1	CKD	6.089	0.075	5.942	6.237
2	CKD	5.942	0.076	5.793	6.091
3	CKD	5.635	0.076	5.486	5.784
